# Arm Joint Coordination of Collegiate Basketball Athletes and Recreational Players when Shooting behind the 3-Point Line

**DOI:** 10.5114/jhk/203104

**Published:** 2025-04-30

**Authors:** Jiaying Li, Youngsuk Kim, Han Li, Bin Zhu, Sukwon Kim

**Affiliations:** 1 Department of Physical Education, Jeonbuk National University, Jeonju, Republic of Korea.; 2 Department of Physical Education, Anhui Normal University, Wuhu, China.

**Keywords:** inter-joint, angle, CAV, shooting mechanics

## Abstract

The primary purpose of this study was to identify exemplary basketball shooting mechanics to devise an effective and efficient training method for successful basketball shooting. Motion data of basketball shots by 10 recreational players and 10 college athletes at three different distances (3.2 m, 5 m, 6.8 m) were collected using 13 cameras (240 Hz). The present study quantified the upper extremity joint coordination using the vector-coded Coupled Angular Variability (CAV). Recreational players exhibited higher CAV at 6.8 m (Median P50 = 16.947), whereas college athletes exhibited higher CAV at 5 m (Median P50 = 18.487). The primary focus of arm coordination patterns was on shoulder joint coordination during the preparation phase, with higher coordination variability associated with greater shot accuracy. Recreational athletes straightened their shoulder and elbow joints simultaneously when performing a basketball shot. In contrast, collegiate athletes showed variations primarily resulting from proximal coordination patterns, leading to a larger range of motion (ROM) for elbow joint flexion and extension. This finding could fundamentally alter how shooting is practiced.

## Introduction

Basket shooting is an important offensive skill that directly affects games. Players use other basketball offensive skills, such as dribbling and passing, to create optimal shooting positions. Surveys of game-related statistics show that effective shooting percentages (as well as defensive rebounds, free-throw attempts, and assistance) correlate with winning and losing elite basketball games (Ibáñez et al., 2009; Lago-Peñas et al., 2010; Lorenzo et al., 2010). Except for jump shots, all other shooting styles were primarily focused on the basket. More than 60% of Women’s National Basketball Association (WNBA) shot attempts during the 2010 season were jump shots (Oudejans et al., 2012). During the 2022–2023 season of the National Basketball Association (NBA), more than 50% of scoring of all teams came from jump shots (*Teams Shooting*, [(accessed on 22 October 2023)]). These seasonal statistics illustrate the importance of jumping shots in basketball.

Several studies have found that players who can shoot from different distances are more dominant in competitive games, especially in the final minutes of evenly matched games, where hitting a long-range jumper proves to be the game-winner (Ardigò et al., 2018). However, jump-shot tasks become more difficult to perform as the shooting distance increases. Simultaneously, the accuracy of the shot decreases, as this greater constraint places higher demands on the player's control strategy for their jump-shot movement. Examples include muscle strength, coordination, and fine motor control (Cortis et al., 2011; Marques et al., 2023; Podmenik et al., 2021). Despite their shooting skills, some athletes struggle to adapt to distances. Therefore, it is necessary to understand the adjustment mechanism when players shoot jump shots at different distances, which would help players and coaches develop new ideas for adjusting the shooting techniques of novice and youth athletes.

A basketball jump shot is a complex technical move. Current research on the sports biomechanics of jump shots focuses on examining the characterization of jump-shot movement, mainly in terms of kinematics (Ammar et al., 2016; Cabarkapa et al., 2021a, 2022, 2023; Kambič et al., 2022; Podmenik et al., 2021): positioning of the elbow, flexion of the trunk, and range of motion of the knee. Although these studies collectively provide information on the activity of each joint that influences the effectiveness of a basketball jump shot, the interactions and continuous changes between the joints are not well understood.

Experienced basketball players typically exhibit joint coupling with changes in the kinetic chain from the start of shot preparation to the release of the ball. Specifically, the joint positions at the elbow and the wrist change simultaneously with the release of the ball (Kambič et al., 2022; Podmenik et al., 2021; Robins et al., 2006). This joint position change of the two neighboring joints can be described as an important synergistic relationship between the two joint angles. Some studies have investigated the changes in elbow and wrist coordination during basketball shooting (Mullineaux and Uhl, 2010; Robins et al. 2006). Much of the focus has been on the distal joints, leaving a gap in the understanding of the role of the proximal joints such as the shoulder and the elbow. Proximal-to-distal coordination is crucial for effective movement patterns, making the investigation of the coordination between the shoulder and elbow joints particularly valuable. The shoulder plays a vital role in generating the initial momentum, which is then transferred from the elbow to the wrist. For example, as mentioned in a previous study on baseball throwing, the angular velocity of the distal segment of the arm originates primarily in the proximal phase (Hirashima et al., 2008). Since the angular velocity of the wrist is largely derived from the angular velocities of the shoulder and the elbow, a significant synergistic or coupling relationship exists between the proximal and distal parts of the arm. This relationship is particularly noteworthy because it influences the overall variability of athletes’ movements, which in turn affects their scoring performance (Rosenkranz and Rothwell, 2012; Srinivasan and Mathiowetz, 2006). This suggests that synergy can be explored using coordination-variability, quantified as angle-angle variability between the trials. However, despite its importance, there is still a lack of quantitative studies examining the upper limb joint coordination in basketball shooting, particularly those addressing the diversity of coordination patterns. Therefore, it is essential to explore how the coordination patterns of the two neighboring joints, the shoulder and the elbow, may influence motor performance.

Variability in coordination is traditionally analyzed using combined time-series data from two adjacent joints. While this approach has been employed in various sports, such as using the dot product of angular velocity vectors to quantify kicking velocity in taekwondo (Kim et al., 2011) and the continuous relative phase to examine swimmers' body coordination during swimming (Seifert et al., 2010), it remains limited in providing a comprehensive understanding of intersegmental coordination. Therefore, a more objective and quantitative method is required to explore the unique demands of basketball jump shots. Vector coding (VC), which calculates the vector angles (referred to as coupling angles) between adjacent data points using angle-angle plots, offers valuable insights into the dominance of one segment's motion relative to another (Needham et al., 2014, 2015). VC is considered more reliable than traditional methods because it provides detailed information on intersegment coordination, movement dynamics, and the locomotor advantage of one segment over another (Chang et al., 2008; Needham et al., 2014). Additionally, VC is mathematically straightforward because it avoids the need to compute higher derivatives (e.g., angular velocity) or normalize data, making it particularly advantageous for analyzing sports movements and facilitating practical interpretations.

In summary, whether the coordination pattern of the arms affects a player's ability to perform jump shots at different distances is a question we are eager to explore. Therefore, we aimed to quantify the changes in coordination of the arm joints when a player shot at different distances using VC. The purposes of the present study were: 1) to describe the coordination relationship between the shoulder and elbow joints of the upper limb in the sagittal plane during shooting from different distances in players of different skill levels, and 2) to describe the coordination variability and dominant coordination patterns of the two joints when shooting from different distances among players of different skill levels. The study hypothesized that 1) the coordination patterns of the two joints would differ among players of different skill levels and change with increasing shooting distance, and 2) the coordination variability of the two joints would increase with distance, and the dominant coordination patterns would become more pronounced with increasing distance.

## Methods

### Participants

Ten male college basketball athletes (age, 19.5 ± 1.1 years; body height, 189.8 ± 6.3 cm; body mass, 79.8 ± 7.3 kg; training experience, 6.5 ± 1.6 years) from a Division 2 basketball college of the Korea University Basketball Federation (KUBF) and ten recreational basketball players (age, 21.6 ± 1.4 years; body height, 177.4 ± 3.6 cm; body mass, 77.5 ± 9.1 kg; training experience, 0.0 ± 0.0 years) from a regional college from South Korea were selected for this study. Recreational players participated in basketball games 2–3 times per week and had no injuries to the lower extremities or other parts of the body before the study. The sample size was estimated at a minimum of 20 participants, based on α = 0.05, β = 0.8, and an effect size *f* = 0.35. The experimental protocol was approved by the institutional review board of the Jeonbuk National University, Jeonju, Republic of Korea (approval code: JBNU 2022-04-008-002; approval date: 01 April 2022). Before participating in this study, all participants were informed about the procedure and read and signed an informed consent form.

### Protocol

**Figure 1:**
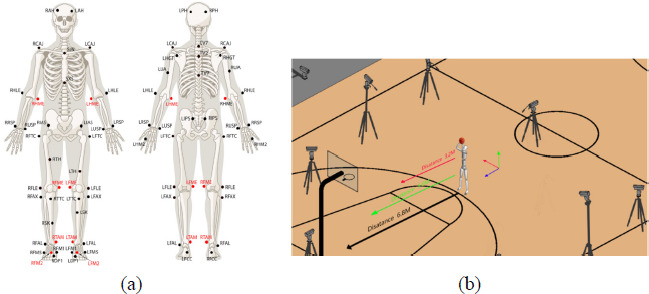
Set up description: (a) marker spot position; (b) experimental environment simulation.

An infrared motion capture system was used to record and synchronize kinematic data during a jump shot by a basketball player. The motion capture system consisted of 13 infrared cameras (OptiTrack, LEYARD, USA) operating at a sampling rate of 240 Hz. In the experiment, reflective markers with a diameter of 14 mm were attached to 57 bony landmarks. Each player had 28 reflective skin markers, including 18 bony markers, six calibration markers, and four markers that distinguished between the left and right thigh and shin segments (Portinaro et al., 2014). Specific information regarding the marker locations is shown in Figure 1(a). The simulation of the experimental environment is shown in Figure 1(b).

**Figure 2:**
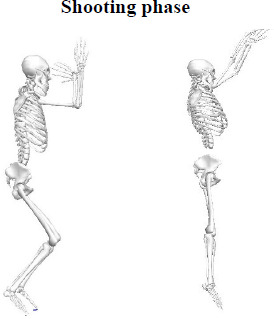
Basketball jump shot action: it starts when the body centre of mass (COM) drops to its lowest point and ends when the ball is released.

Each participant was asked to make jump shots at three different distances (3.2 m, 5 m, 6.8 m), and three successful jump shots were taken for each player (i.e., the shot was considered successful when the ball was thrown into the basket). The phases of the jump-shot motion are shown in Figure 2.

### Data Analyses

Raw data collected by a motion capture system (OptiTrack, LEYARD, USA) was imported by means of Visual 3D software (Experienced 6.0; C-Motion Inc., Germantown, MD, USA). The kinematic data were low-pass filtered using a 4^th^-order zero-lag Butterworth filter with a cut-off frequency of 10 Hz (Saini et al., 2020). Joint angles were calculated from the distal segment relative to the proximal segment using an X-Y-Z Cardan rotation sequence (Augustus et al., 2021; Choi et al., 2016). This study focused on analyzing joint angle information in the sagittal plane. All players shot right-handed in their jump shots; therefore, it was also information on the players’ dominant side (right side).

The coupling angles of the two neighboring joints in the sagittal plane were obtained by calculations using MATLAB software (version R2022b; Math Works Inc., Natick, MA, USA) with a modified vector code (Needham et al., 2014). In addition, all the sagittal plane angles were temporally normalized to 100% of the entire cycle before calculating the coupling angles.

### Calculation of the coupling angle and coordination variability

For each instant (i) during the shooting phase, the coupling angle (γ_i_) was calculated based on the consecutive proximal segmental angles ( θ_P(i)_, θ_P(i+1)_ ) and consecutive distal segmental angles ( θ_D(i)_, θ_D(i+1)_) according to Eqs. (1) and (2) as follows:


(1) $${\text{γ}}_{\text{i}}=\text{Atan}\left(\frac{{\text{θ}}_{\text{D}(\text{i}+1)}-{\text{θ}}_{\text{Di}}}{{\text{θ}}_{\text{P}(\text{i}+1)}-{\text{θ}}_{\text{Pi}}}\right)\frac{180}{\text{π}}\;({\text{θ}}_{\text{P}(\text{i}+1)}-{\text{θ}}_{\text{Pi}}>0)$$

(2) $${\text{γ}}_{\text{i}}=\text{ Atan}\left(\frac{{\text{θ}}_{\text{D}(\text{i}+1)}-{\text{θ}}_{\text{Di}}}{{\text{θ}}_{\text{P}(\text{i}+1)}-{\text{θ}}_{\text{Pi}}}\right)\frac{180}{\text{π}}+180\;({\text{θ}}_{\text{P}(\text{i}+1)}-{\text{θ}}_{\text{Pi}}<0)$$

The following conditions (3) were applied:


(3) $${\text{γ}}_{\text{i}}\;=\left\{\begin{array}{c} {\text{γ}}_{\text{i}}=90\;({\text{θ}}_{\text{P}(\text{i}+1)}-{\text{θ}}_{\text{Pi}}=0\;and\;{\text{ θ}}_{\text{P}(\text{i}+1)}-{\text{θ}}_{\text{Pi}}>0)\\ {\text{γ}}_{\text{i}}=-90\;({\text{θ}}_{\text{P}(\text{i}+1)}-{\text{θ}}_{\text{Pi}}=0\;and\;{\text{θ}}_{\text{P}(\text{i}+1)}-{\text{θ}}_{\text{Pi}}<0)\\ {\text{γ}}_{\text{i}}=-180\;({\text{θ}}_{\text{P}(\text{i}+1)}-{\text{θ}}_{\text{Pi}}<0\;and\;{\text{θ}}_{\text{P}(\text{i}+1)}-{\text{θ}}_{\text{Pi}}=0)\\ {\text{γ}}_{\text{i}}=Undefined\;({\text{θ}}_{\text{P}(\text{i}+1)}-{\text{θ}}_{\text{Pi}}=0\;and\;{\text{θ}}_{\text{P}(\text{i}+1)}-{\text{θ}}_{\text{Pi}}=0) \end{array}\right.$$

The coupling angle (γ_i_) was corrected to present a value between 0°and 360° according to Eq. (4):


(4) $${\text{γ}}_{\text{i}}\;=\left\{\begin{array}{c} {\text{γ}}_{\text{i}}+360\;({\text{γ}}_{\text{i}}<0)\\ {\text{γ}}_{\text{i}}\;({\text{γ}}_{\text{i}}\geq 0) \end{array}\right.$$

Since the calculated coupling angles (γ_i_) were directional and originated from the interval range of 0°–360°, the use of a series of arithmetic averages within the action phases would lead to errors in the averages and would not represent the correct orientation of the vectors. Therefore, the average coupling angle (γ̄_l_) and coordination variability (CAV_i_) were calculated using circular statistics (Batschelet, 1981; Hamill et al., 2000).

The average coupling angle (γ̄_l_) was calculated based on the average horizontal (x̄_l_) and vertical (ȳ_l_) components at each instant using circular statistics (5) and (6) as follows:


(5) $$\bar{{\text{x}}_{\text{i}}}=\frac{1}{\text{n}}\overset{\text{n}}{\underset{\text{i}=1}{\sum }}\cos{\text{γ}}_{\text{i}}$$

(6) $$\bar{{\text{y}}_{\text{i}}}=\frac{1}{\text{n}}\overset{\text{n}}{\underset{\text{i}=1}{\sum }}\sin{\text{γ}}_{\text{i}}$$

The following Eq. (7) was applied to correct for the average coupling angle (γ̄_l_) to present a value between 0°and 360°.


(7) $$\bar{{\text{γ}}_{\text{i}}}=\left\{\begin{array}{c} Atan\left(\frac{\bar{{\text{y}}_{\text{i}}}}{\bar{{\text{x}}_{\text{i}}}}\right)\frac{180}{\text{π}}\;({\text{x}}_{\text{i}}>0,{\text{y}}_{\text{i}}>0)\\ Atan\left(\frac{\bar{{\text{y}}_{\text{i}}}}{\bar{{\text{x}}_{\text{i}}}}\right)\frac{180}{\text{π}}+180\;({\text{x}}_{\text{i}}<0,)\\ Atan\left(\frac{\bar{{\text{y}}_{\text{i}}}}{\bar{{\text{x}}_{\text{i}}}}\right)\frac{180}{\text{π}}+360\;({\text{x}}_{\text{i}}>0,{\text{y}}_{\text{i}}<0)\\ 90\;({\text{x}}_{\text{i}}=0,{\text{y}}_{\text{i}}>0)\\ -90\;({\text{x}}_{\text{i}}=0,{\text{y}}_{\text{i}}<0)\\ Undefined\;({\text{x}}_{\text{i}}=0,{\text{y}}_{\text{i}}=0) \end{array}\right.$$

The length of the average coupling angle (r̄_l_) was calculated according to Eq. (8):


(8) $$\bar{{\text{r}}_{\text{i}}}=\sqrt{{\bar{{\text{x}}_{\text{i}}}}^{2}+{\bar{{\text{y}}_{\text{i}}}}^{2}}$$

Coupling angle variability (CAV_i_) was calculated according to Eq. (9):


(9) $${\text{CAV}}_{\text{i}}=\sqrt{2(1-\bar{{\text{r}}_{\text{i}}})}\frac{180}{\text{π}}$$

### Statistical Analyses

All statistical analyses were conducted using IBM SPSS statistics software (International Business Machines Corp., Armonk, NY, USA). Initially, Coupled Angular Variability (CAV) for each distance was assessed for normality using the Kolmogorov-Smirnov test, assuming that the data were normally distributed. The results indicated *p*-values less than 0.05, suggesting rejection of the null hypothesis owing to the non-normal distribution of the data. Consequently, non-parametric tests were used for all group comparisons. Appropriate non-parametric tests were used to analyze differences across distances in cases of unequal variances or non-normal distributions. Specifically, the Mann-Whitney test was used to compare players of different performance levels, and the Friedman test was applied to analyze differences in joint coupling angle variability (CAV) across the three distances. Post-hoc comparisons were conducted using the paired Wilcoxon signed-rank test with Bonferroni correction (N = 3, *p* < 0.017 for significant differences). Significant findings were further described by comparing the median values (M P_25_ and P_75_).

Comparisons of frequency distributions of coordination patterns both between and within groups were conducted using the chi-squared test. Between-group distribution comparisons were used to analyze the relationships between the frequency distributions of coordination patterns among different skill levels at the same distance. Within-group distribution comparisons were used to examine the relationships between the coordination pattern frequencies across distances within the same skill level. The chi-square test measured the degree of deviation between the observed and expected frequencies. A larger chi-square value indicated greater deviation, whereas a smaller value suggested a closer fit to the expected distribution. A chi-square of zero indicated that the frequency distributions of the two groups were very similar. Categorical data were considered for each variable, and chi-square values along with the corresponding *p*-values were used to determine the presence of differences. Significant differences were indicated at *p* < 0.01.

## Results

### Illustration Description

**Figure 3:**
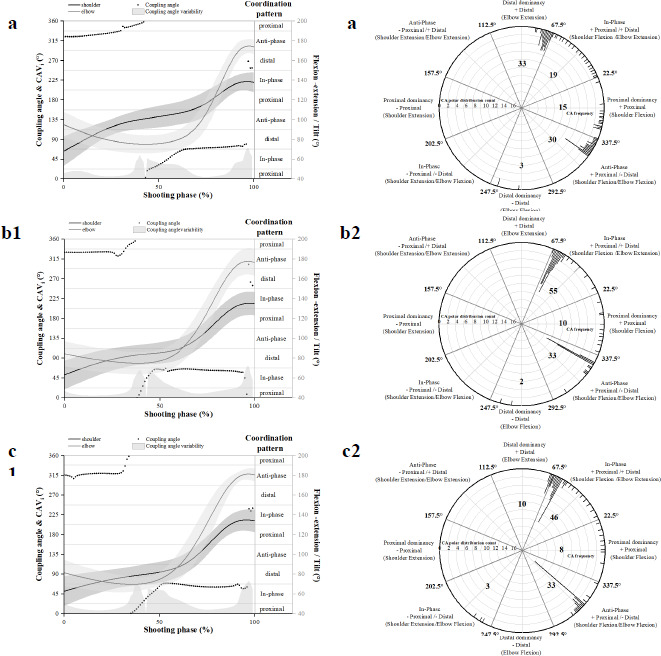
Mean coupling angles for shoulder-elbow coordination in the sagittal plane during a basketball jump shot of recreational players are presented using raw illustrations and classification of coordination patterns. All firing phases are time-variant and are normalized to a percentage (0–100%) of the firing phase time.

**Figure 4:**
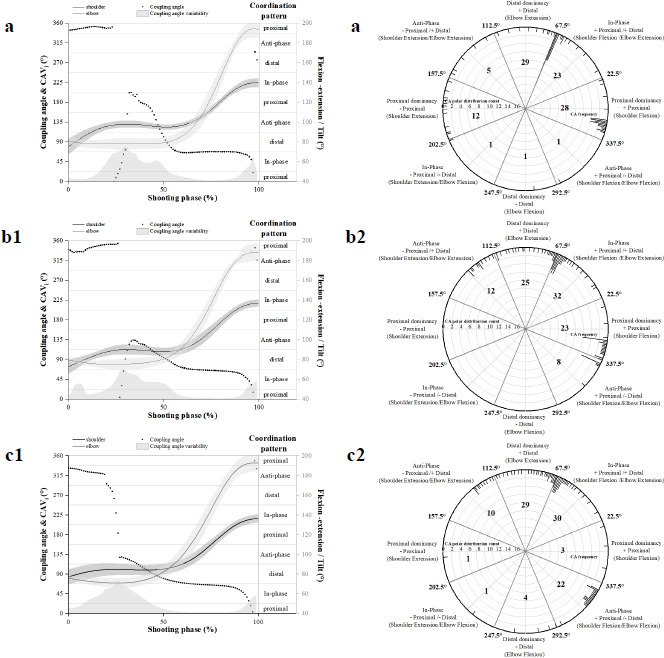
Mean coupling angles for shoulder-elbow coordination in the sagittal plane during a basketball jump shot of college athletes are presented using raw illustrations and classification of coordination patterns. All firing phases are time-variant and are normalized to a percentage (0–100%) of the firing phase time.

Figures 3 and 4 demonstrate the coordination patterns on the sagittal plane of the shoulder and elbow joints (a indicates shooting at 3.2 m; b indicates shooting at 5 m; c indicates shooting at 6.8 m.) The sagittal plane angle data of the two adjacent joints for the three distance-firing phases are represented by black and gray solid lines, and the associated sagittal plane ROM change information is located on the right vertical axis of the graph. The black dot information in the figure is the calculated mean coupling angle (γ̄_l_): it represents the coordination pattern of the sagittal planes of the two adjacent joints (proximal motion, distal motion, in-phase motion, anti-phase motion), and indicates the co-ordination relationship between the angular data of the two adjacent joints throughout the firing phase. The grey shaded area at the bottom of the inset represents variability in the coupling angles (CAV_i_). Both quantitative metrics were quantified in degrees, and the relevant information is shown on the vertical axes on the left and right sides of the figure.

In all frequency distribution charts of the coordination patterns (Figure 3), the calculated average coupling angles were incorporated into the polar coordinate plots. Within each polar plot, based on the activities in the two directions of the sagittal plane, we categorized the coordination patterns of adjacent joint planes into eight categories. These categories were assigned to intervals on the polar plot according to the coupling angle (as noted in the chart annotations). Each interval was counted to determine the frequency distribution of each coordination pattern. Frequency distributions were quantified by counting and are displayed around the periphery of the polar plots.

### Patterns of Coordination and Frequency Distribution between Two Joints

Tables 1–3 show the differences in coordination variability between the shoulder and elbow joints in the sagittal plane, as well as their dominant coordination patterns when players shot at three different distances.

**Table 1:** Comparison of coupled angular variability (CAV) of the shoulder and elbow joints.

	Median values			Friedman test
	3.2 m	5 m	6.8 m	Statistical value (*p*-value)
Recreational players	16.078(7.8,33.8) ^bc^	15.093(8.8,29.8) ^c^	16.947(10.4,40.6) ^Aa^	31.920 (<0.001)
College athletes	15.859(6.3,44.9) ^b^	18.487(4.7,38.7) ^ac^	14.654(3.4,39.7) ^Bc^	31.760 (<0.001)

Post-hoc comparisons were performed using the Paired Wilcoxon signed-rank test with Bonferroni correction applied. A p-value < 0.017 indicates significant difference. Differences between groups (p < 0.05) are denoted by different uppercase letters, while differences within groups (p < 0.05) are indicated by different lowercase letters. Specifically, uppercase letters A and B signify a significant difference between recreational players and college athletes, whereas lowercase letters a, b, and c denote significant differences among the three distances. A: significantly different from recreational players, B: significantly different from college athletes; a: significantly different from 3.2 m, b: significantly different from 5 m. c: significantly different from 6.8 m

**Table 2:** Comparison of frequency distributions of shoulder and elbow coordination patterns in recreational basketball players.

	Frequency distributions			Chi-square (*p*-value)		
	3.2 m	5 m	6.8 m	3.2 m vs. 5 m	3.2 m vs. 6.8 m	5 m vs. 6.8 m
proximal-dominance (shoulder flexion)	15	10	8	1.143(0.285)	2.407 (0.121)	0.244 (0.621)
in-phase (shoulder flexion/elbow extension)	19	55	46	27.799(<0.001)	16.615(<0.001)	1.620 (0.203)
distal-dominance (elbow extension)	33a	0	10	39.521(<0.001)	15.672(<0.001)	10.526(0.001)
anti-phase (shoulder extension/elbow extension)	0	0	0	____	____	____
proximal-dominance (shoulder extension)	0	0	0	____	____	____
in-phase (shoulder extension/elbow flexion)	0	0	3	____	3.046 (0.246)	3.046 (0.246)
distal-dominance (elbow flexion)	3	2	0	0.205(1.000)	3.046 (0.246)	2.020 (0.497)
anti-phase (shoulder flexion/elbow flexion)	30	33	33	0.209(0.648)	0.209 (0.648)	____

χ^2^ represents the chi-square value. The larger the chi-square value, the greater the deviation from the expected distribution; the smaller the chi-square value, the closer the data are to the expected distribution. A p-value < 0.05 indicates a significant difference in the data. The shaded areas in the table indicate between-group differences, while within-group differences are represented by p-values

**Table 3:** Comparison of frequency distributions of shoulder and elbow coordination patterns in the experienced group of college basketball players.

	Frequency distributions			Chi-square (*p*-value)		
	3.2 m	5 m	6.8 m	3.2 m vs. 5 m	3.2 m vs. 6.8 m	5 m vs. 6.8 m
proximal-dominance (shoulder flexion)	28	23	3	0.658 (0.417)	23.860 (<0.001)	17.683 (<0.001)
in-phase (shoulder flexion/elbow extension)	23	32	30	2.031 (0.154)	1.258 (0.262)	0.094 (0.760)
distal-dominance (elbow extension)	29	25	29	0.406 (0.524)	____	0.406 (0.524)
anti-phase (shoulder extension/elbow extension)	5	12	10	3.150 (0.076)	1.802 (0.179)	0.204 (0.651)
proximal-dominance (shoulder extension)	12	0	1	12.776 (<0.001)	9.955 (0.002)	____
in-phase (shoulder extension/elbow flexion)	1	0	1	____	____	____
distal-dominance (elbow flexion)	1	0	4	____	1.846 (0.369)	4.082 (0.121)
anti-phase (shoulder flexion/elbow flexion)	1	8	22	5.701 (0.035)	21.665 (<0.001)	7.686 (0.006)

χ^2^ represents the chi-square value. The larger the chi-square value, the greater the deviation from the expected distribution; the smaller the chi-square value, the closer the data is to the expected distribution. A p-value < 0.05 indicates a statistically significant difference in the data. The shaded areas in the table indicate between-group differences, while within-group differences are represented by p-values

From Table 1, it can be observed that at a distance of 6.8 m from the jump shot, there was a significant difference in CAV between recreational and college athletes (Mann-Whitney z = 2.111, *p* = 0.035). It is evident that recreational shooters had higher CAV at a distance of 6.8 m (Median P_50_ = 16.947), with significant differences compared to CAV at 3.2 m (Statistical z = 4.999, *p* = 0.000) and 5 m (Statistical z = 5.422, *p* = 0.000). College athletes exhibited higher CAV at 5 m (Median P_50_ = 18.487), with significant differences compared to CAV at 3.2 m (Statistical z = 4.556, *p* = 0.000). Simultaneously, there were also differences in CAV between 3.2 m and 6.8 m (Statistical z = 2.816, *p* = 0.005).

Recreational players exhibited more distal-dominance coordination patterns when shooting at 3.2 m, with significant differences compared to the coordination patterns at 5 m (χ^2^ = 39.521, *p* = 0.000) and 6 m (χ^2^ = 15.672, *p* = 0.000) (Table 2). At a distance of 5 m and 6.8 m, there were relatively more in-phase coordination patterns, with significant differences compared to the coordination patterns at 3.2 m (χ^2^ = 27.799, *p* = 0.000; χ^2^ = 16.615, *p* = 0.000). College athletes exhibited more proximal-dominance coordination patterns when shooting at 3.2 m, with significant differences compared to the coordination patterns at 5 m (χ^2^ = 12.776, *p* = 0.000) and 6 m (χ^2^ = 23.860, *p* = 0.000) (Table 3). At a distance of 5 m, there were relatively more in-phase and proximal-dominance coordination patterns, with significant differences compared to the proximal-dominance patterns at 6 m (χ^2^ = 17.683, *p* = 0.000). At 6.8 m, there were relatively more distal-dominance and anti-phase coordination patterns, especially in the anti-phase dominant patterns, showing significant differences compared to 6 m (χ^2^ = 21.665, *p* = 0.000).

## Discussion

The present study utilized vector coding analysis to quantify the coordination between the adjacent shoulder and elbow joints in athletes of different performance levels executing jump shots at three different distances. Recreational players exhibited the greatest coupled angular variability (CAV) in shoulder-elbow coordination at a distance of 6.8 m, while the smallest CAV occurred at 5 m. Conversely, college athletes showed the largest CAV at 5 m and the smallest at 6.8 m. In addition, recreational players displayed variations primarily arising from in-phase coordination patterns across different shooting distances, adopting the strategy of simultaneous movement in the same direction for the shoulder and elbow joints in the sagittal plane. Conversely, college athletes showed variations that primarily stemmed from proximal coordination patterns. Consistent with our hypothesis, the coordination patterns of the two joints changed with the player's skill level and increasing shooting distance; however, contrary to expectations, the coordination variability between the two joints did not vary with increases in the players’ performance level and shooting distance. Unexpectedly, the present study found that the trend of CAV changes differed among players of different performance levels, with college athletes exhibiting higher CAV changes primarily during the preparatory phase of shooting. Another finding was that the dominant coordination patterns changed with increasing distance; however, the trends were similar among players of the same skill level.

Although some studies have demonstrated that higher shooting accuracy correlates with greater coordination variability (Mullineaux and Uhl, 2010), performance of recreational players contradicts this perspective: the present study found that recreational players did not have high accuracy in the 6.8-m jump shot, although coordination variability was high. This may be due to the increased difficulty of the task at 6.8 m, leading to uncontrollable variations in the movement patterns of the shoulder and elbow joints due to a lack of experience. In contrast, college athletes validated this concept: during 5-m jump shots, both shooting accuracy and coordination variability were higher than at other distances, which aligned with findings by Okazaki and Rodacki (2012) on shooting accuracy. The 5-m jump shot position was close to the free-throw line, where players, during games and practice, showed a higher probability of scoring than when performing long-distance shots. However, close-range shots are not common in games because of the defensive pressure near the basket, leading players to opt more often for lay-ups or dunks (Oudejans et al., 2012) to score effectively. This indicates that variability in shooting coordination is not affected by shooting distance, but is related to the success rate of free throws.

This is attributed to variability being assumed to be a compensatory strategy for continual adjustments to prevent technical errors (Pakosz et al., 2021), a strategy made possible by the players' ample experience. However, studies have confirmed that shooting accuracy significantly decreases with increasing shooting distance, and players require a greater range of motion in the arm joints to increase the release speed, especially a greater range of motion in the shoulder (Okazaki et al., 2004; Okazaki and Rodacki, 2012). Despite different shooting distances, college athletes compensate for movement deficiencies to achieve accurate shots through compensatory strategies, primarily from the proximal to the distal joints, especially with larger adjustments in both the proximal and distal joints. College athletes maintain consistent hand and forearm angular velocities and accelerations across shots (Okubo and Hubbard, 2020). Our study validates this point: as distance increased, experienced basketball players made larger adjustments in the shoulder during the early shooting phase, followed by the forearm, and then released the ball through the wrist. In particular, at each distance, college athletes exhibited higher proximal coordination modes than recreational players.

This adjustment strategy was confirmed in the present study: college athletes showed greater early adjustments in the shoulder joint relative to the elbow joint as shooting distance increased because the angular velocity and acceleration of the upper arm were crucial for release conditions (Okubo and Hubbard, 2020). Furthermore, research on the regulation of shooting techniques (Bartlett et al., 2007; Button et al., 2003; Robins et al., 2006) has found that the pattern of joint angle changes along the proximal-distal movement chain positively impacts performance and can act as a compensatory functional change. Similarly, in a recently published study, forearm positioning was found to be a key kinematic variable capable of distinguishing between proficient and non-proficient free-throw players (Cabarkapa et al., 2021a). For recreational players, as previously mentioned, with the increase in distance, sudden changes occurred in the movement patterns of the shoulder and elbow joints, especially during transitions, while simultaneously increasing the range of motion of both the shoulder and elbow joints, similar to “pushing” the ball towards the basket. This is similar to the findings of Button et al. (2003): players with little experience exhibited increased elbow angular velocity and displacement with increasing distance, yet the maximum angular velocity and range of motion of the elbow joint were lower than those of other players (Button et al., 2003).

Button et al. (2003) discussed motor variability in basketball free-throw shooting with respect to the varying skill levels of female players, including an experienced national team captain, two under-18 national team players, and a minimally experienced player. The skilled performers were characterized by increased inter-trial consistency in the elbow and wrist joints. However, the trajectory variability did not significantly decrease with improved skills. Trajectory variability refers to the standard deviation of the linear elbow displacement at discrete points during the throwing motion. The variability in movement and coordination can be functional, allowing adaptation to environmental or task-specific demands. This functional adjustment facilitates changes in the coordination patterns. Such variations in coordination among athletes challenge the notion of singular optimal movement patterns and techniques. Furthermore, this variability in joint angles at release does not adversely affect the height, the angle or speed of release, suggesting the presence of compensatory mechanisms at the wrist and elbow joints that minimize the variability in projectile release variables, thereby implying a more functional role for movement variability. We believe that skilled performers increase the range of the shoulder joint to accelerate the transition of the throwing arm into the throwing phase. Flexion of the shoulder and elbow joints lifts the ball with continuous adjustments of the shoulder joint, positioning the ball optimally for the throw.

It can be observed that the focus of upper limb coordination patterns is primarily related to greater adjustments in the shoulder joint during the preparatory phase, which also explains why college athletes often seek the optimal position of the upper arm early in their movement (Button et al., 2003). This is contrary to the findings of Cabarkapa et al. (2023) who suggested that shoulder joint flexion should be minimized during the preparatory phase. Second, it relates to the predominance of elbow extension and greater wrist activity as the release of the ball approaches, further elucidating that elbow extension-wrist flexion forces are planned in a feedforward mode based on the planned position of the arm given by shoulder displacement (Okazaki et al., 2008).

A crucial insight from this study is that higher coordination variability appears to correlate with higher shooting accuracy, suggesting that players’ accuracy is best understood as being primarily dependent on their ability to control deviation. Instead, as it was previously misunderstood, movement patterns of skilled athletes remain unchanged. These findings have the potential to fundamentally transform approaches to shooting training. In contrast to traditional training methods that focus on developing highly repeatable motions, training that enhances proprioception between the proximal and distal joints may have a more significant impact on performance.

The present study has important implications for basketball shooting training. First, it was found that higher coordination variability between the shoulder and elbow joints correlated with higher shooting accuracy, particularly among skilled athletes. This challenges the traditional perspective that consistent and repeatable motions are the key to accuracy. Therefore, coaches and players should focus on training that enhances proprioception and the ability to control movement variability rather than strictly repeating identical motions. Second, college athletes demonstrated a strategy of proximal-to-distal coordination, in which greater adjustments in the shoulder joint during the early phase of shooting compensated for movement deficiencies, leading to more accurate shots as the distance increased. Skilled athletes often make early adjustments to shoulder joint positioning during the preparatory phase to optimize their shooting performance. This is crucial for controlling the transition from the arm to the shooting phase. Therefore, training programs should emphasize the importance of developing control over the proximal joints (such as the shoulder) early in the shooting process. Thus, athletes could better manage the compensatory mechanisms required for accurate long-distance shooting. Emphasizing early shoulder positioning in shooting drills can help athletes achieve a more effective ball release and improve overall shooting accuracy. Coaches should integrate exercises that enhance shoulder flexibility and control in movement sequences. Third, this study showed that recreational players exhibited significant changes in movement patterns with increasing distance, particularly the tendency to “push” the ball over longer distances, leading to lower accuracy. In contrast, skilled athletes maintained consistent coordination patterns across different distances. Therefore, coaches should tailor training drills to address the challenges of long-distance shooting in less experienced players, focusing on stabilizing shoulder and elbow movements to prevent the tendency to push the ball. This can help improve performance at various shooting ranges. Lastly, the study observed that coordination patterns and variability changed depending on shooting distance, with college athletes showing distinct patterns at 5 m versus 6.8 m. Therefore, coaches should design shooting drills tailored to different distances, focusing on the specific coordination patterns that are most effective for each range. This approach can help players develop appropriate motor skills and coordination strategies required for varying in-game scenarios.
